# Sorting Insiders From Co-Workers: Remote Synchronous Computer-Mediated Triage for Investigating Insider Attacks

**DOI:** 10.1177/00187208211068292

**Published:** 2022-03-07

**Authors:** Coral J. Dando, Paul J. Taylor, Tarek Menacere, Thomas C. Ormerod, Linden J. Ball, Alexandra L. Sandham

**Affiliations:** Department of Psychology, University of Westminster 4921 , London; Department of Psychology, Lancaster University 4396 , Lancaster, UK; School of Psychology, University of Sussex 1948 , Falmer, UK; School of Psychology, University of Central Lancashire 6723 , Preston, UK; Department of Psychology, University of Gloucestershire 2376 , Cheltenham, UK

**Keywords:** insiders, computer-mediated triage, deception, investigation

## Abstract

**Objective:**

Develop and investigate the potential of a remote, computer-mediated and synchronous text-based triage, which we refer to as *InSort,* for quickly highlighting persons of interest after an insider attack.

**Background:**

Insiders maliciously exploit legitimate access to impair the confidentiality and integrity of organizations. The globalisation of organisations and advancement of information technology means employees are often dispersed across national and international sites, working around the clock, often remotely. Hence, investigating insider attacks is challenging. However, the cognitive demands associated with masking insider activity offer opportunities. Drawing on cognitive approaches to deception and understanding of deception-conveying features in textual responses, we developed InSort, a remote computer-mediated triage.

**Method:**

During a 6-hour immersive simulation, participants worked in teams, examining password protected, security sensitive databases and exchanging information during an organized crime investigation. Twenty-five percent were covertly incentivized to act as an ‘insider’ by providing information to a provocateur.

**Results:**

Responses to InSort questioning revealed insiders took longer to answer investigation relevant questions, provided impoverished responses, and their answers were less consistent with known evidence about their behaviours than co-workers.

**Conclusion:**

Findings demonstrate InSort has potential to expedite information gathering and investigative processes following an insider attack.

**Application:**

InSort is appropriate for application by non-specialist investigators and can be quickly altered as a function of both environment and event. InSort offers a clearly defined, well specified, approach for use across insider incidents, and highlights the potential of technology for supporting complex time critical investigations.

## Introduction

Insiders exploit privileged access to damage organizations (see Mills et al., 2017; Posey et al., 2013). Examples include a BUPA employee who downloaded and offered for sale 547,000 items of patient information and a NASA employee who downloaded classified national defence information. Insider crime is increasing (Homoliak et al., 2019; Clearswift Insider Threat Index, 2017) and becoming more expensive (European Union Agency for Cybersecurity, 2020; National Law review, 2020). Surveys suggest 27% of cybercrime incidents are committed by insiders (Trzeciak, 2019) with insiders responsible for 43% of data loss reported by the world’s largest companies (Intel Security, 2015). Insider threats are difficult to mitigate. Employees are trusted, with detailed knowledge and access to employer assets. Understanding of insider behaviours and psychological characteristics is improving (e.g., Costa, et al., 2016; Elmrabit et al., 2020; Greitzer et al., 2018; Spitzner, 2003; Taylor et al., 2013). However, few insider investigative techniques exist (Maybury, 2006) because knowledge derived from one attack is not necessarily relevant to others (e.g., CPNI, 2020; Saxena et al., 2020).

### Computer-Mediated Triage

Gathering post attack information is fundamental to understanding what has happened. In doing so, investigators (in-house security or external agencies) seek to understand the veracity of employee accounts. Employees may be dispersed across numerous national or international sites and so conducting timely and effective investigations can be challenging. Here, we evaluate text-based computer-mediated communication (CMC) using a series of event-specific questions towards meeting this challenge. Computer-mediated communication screening is increasingly used to support decision-making where there are high volumes of traffic such as for pre-screening job applicants and completing employee credibility assessments (Jenson et al., 2013; Twyman et al., 2014). Building on research concerning the language of insiders (Jenkins & Dando, 2012; Taylor et al., 2013), we investigated whether synchronous textual responses to CMC questions might effectively triage persons of interest.

Computer-mediated communication has several potential advantages. Organizations can gather information from employees simultaneously, irrespective of location, offering speed, volume, and reach (e.g., Lew et al., 2018; Pang et al., 2018; Yao & Ling, 2020). Text-based CMC is widely accessible, technically stable and is low in media richness and so devoid of non-verbal cues that occur during face-to-face interactions that can negatively impact investigations, potentially reducing false positives and negatives (e.g., Bond & DePaulo, 2006; Dando & Ormerod, 2017; Markowitz, 2020; Matsumoto et al., 2011; Meissner & Lyles, 2019; Nortje & Tredoux, 2019; Walsh et al., 2018).

### Masking Malicious Behaviour

Psychological knowledge of the challenges of masking malicious activity offers strategic insight into how to structure a CMC triage. To remain above suspicion necessitates deceiving colleagues (e.g., Homoliak et al., 2019; Lew et al., 2018; Taylor et al., 2013). Hence, insiders have an impression management goal (L. H. Colwell et al., 2006; Weiss & Feldman, 2006). They have to provide deceptive accounts that appear truthful and so have to manage ‘two employment worlds’: tasks they should and should not have completed. Hence, providing a convincing false account is more demanding than completing legitimate activity and then providing a truthful account. This disparity offers opportunities for detection (e.g., K. Colwell et al., 2007; Kohan et al., 2020; Vrij et al., 2017).

Increased cognitive load in such circumstances (e.g., Bhatt et al., 2009; Jiang et al., 2015) can result in differential verbal behaviours between liars and truthtellers. Liars often provide less consistent or coherent verbal accounts lacking informational content, with fewer event details (Bogaard et al., 2016; DePaulo et al., 2003; Hartwig et al., 2011). Differences can be enhanced by tactical questioning techniques (e.g., Blandon-Gitlin et al., 2014; Dando & Bull, 2011; Dando & Ormerod, 2020; Hamlin et al., 2020; Ormerod & Dando, 2015; Sporer, 2016; Vrij et al., 2010), which have yielded over 70% accuracy where the base rate of deceivers was just 1:1000 (Dando & Ormerod, 2020; Ormerod & Dando, 2015), compared with a typical detection rate of 54% (e.g., Bond & DePaulo, 2006; Hauch et al., 2016). Similar results are reported in laboratory-based research (e.g., Dando & Bull, 2011; Granhag & Hartwig, 2015; Levine, 2014; Sandham et al., 2020).

Detecting deception via tactical questioning is largely situated in face-to-face and media-rich interview contexts. Nonetheless, several techniques lend themselves to CMC triage with potential for leveraging measurable indicators of deception (Lee et al., 2009; Zhou et al., 2004), particularly where comparisons can be made across employee responses gathered following each insider attack (Burgoon et al., 2003; Rubin et al., 2015). For example, deception-conveying features can sometimes include wordy replies with low information (e.g., Pollina et al., 2017; Vendemia et al., 2005) and more expressions of uncertainty (Zhou et al., 2004).

### Towards a Solution

Combining cognitive approaches to deception and understanding of deception-conveying features in textual responses, we developed a novel CMC text-based triage: *InSort* (**In**sider **Sort**). InSort comprised a series of bespoke questions dictated by the insider event itself, the run-up to the event, and workers day-to-day work activities (e.g., necessary, unnecessary and not allowed). Additionally, various questioning strategies were employed. Target questions concern attack-specific behaviours, including behaviours in the run up to an attack, questions about attempted access to databases, physical movements and communication. Target questioning increases cognitive complexity for insiders to maximize the collection of triage-relevant information. Open questions (tell, explain, describe) gather accounts about specific times, necessitating provision of expansive answers. These question types and their tactical presentation makes it challenging for insiders to provide a coherent account (e.g., Dando & Bull, 2011; Dando & Ormerod, 2020; Ormerod & Dando, 2015).

Target questions are manipulated to impose high cognitive demands on liars. They are not presented *en bloc* nor chronologically, thereby introducing a temporal element (requiring maintenance of six worlds – true and false versions of past, present and future). Some target questions are repeated, accentuating between-question inconsistencies and contradictions, which can be indicative of deceit (Blair et al., 2018; Chan & Bull, 2014; Vredeveldt et al., 2014). Responses are required before moving to the next question. Thus, InSort is interactive (e.g., Lee et al., 2009; Sánchez-Junquera et al., 2020; Zhou et al., 2004), demanding higher levels of cognitive engagement (Burgoon et al., 2010). The immediacy of InSort reduces opportunities to construct deceptive accounts or confer with accomplices versus lengthier triage processes conducted by human investigators (Levine et al., 2018; Walczyk et al., 2013).

In sum, InSort may confer advantages including speed of implementation and increased concurrent cognitive demand for insiders (deceivers), which may leverage deception-conveying features (e.g., Bhatt et al., 2009; Jiang et al., 2015). We conducted a ‘serious gaming’ empirical study, whereby participants were immersed in a full-day office-based collaborative investigations of organized crime. The game, known as Confidential Operations Simulation (iCOS: see Taylor et al., 2013), was played over a series of competitive rounds. To establish a behavioural baseline, the first round was played with no insider. In subsequent rounds, team members were assigned the role of ‘insider’, receiving financial incentives to undertake illicit activities and not to be caught (see Method). The study tested a series of hypotheses:

- Insiders will take significantly longer than non-insiders to complete InSort (H^1^) because of the dual impacts of tactical questioning and limited time to develop lie scripts.
- Impression management will result in insider’s text responses to open target questions being shorter and with less information than non-insiders (H^2^).
- Insiders will be less consistent in their responses to closed target questions, making answer-evidence errors (H^3^).
- Insiders will report finding InSort cognitively demanding and will be less confident in their responses (H^4^).

## Method

### Participants and Procedure

Sixty participants were paid £50 to take part in iCOS games lasting between 6 and 9 hours (*M* = 6.8 hours) – 26 males (*M*_age_ = 25.67, range 18–40 years), and 34 females (*M*_age_ = 23.8 years, range 19–30 years). Each game was split into four rounds and comprised 12 players, randomly assigned to a team (i) Fraud; (ii) Human Trafficking and (iii) Narcotics. Each team comprised four roles: Administrator, Field Agent, Intelligence Analyst and Tactical Investigator. Status and responsibilities within teams were equal.

Teams had to solve a series of linked crimes, one in each round. Teams were presented with intelligence updates about criminal gangs and used this information to guide their searches of password-protected databases. Team players pieced together information to identify gang members and their location. Players’ database access was limited, so team members worked together, exchanging information, recognizing connections across databases, and engaging in collaborative problem solving. The team that most quickly identified and located criminals ‘won’. Teams were financially incentivized to win each round (an additional £20 for winning the round). Each round lasted approximately 90 minutes including regular breaks.

At the start, participants were randomly assigned to a team role. They received instructions about the tasks to be completed, training on using the investigative databases, and familiarized themselves with the databases. To simulate a secure environment, players worked in ‘silent’ offices, making notes using desktop publishing and spreadsheet software and exchanging information using email, SMS messaging and mobile phone conversations. They had access to a printer in a separate room. Once familiar with the environment, one team member received instructions about the first crime to be investigated. All further interaction with participants was conducted via email with ‘Gold Command’ (a confederate). Gold Command issued instructions for subsequent tasks. By embedding task instructions into the simulation, we hoped to enhance participants’ immersion in the simulation (Druckman, 2005).

Prior to the second round (at the end of the first round), up to two players in each team were covertly approached to provide information to a provocateur for an additional £20 reward. Specifically, to obtain information concerning an individual under investigation, and to email this information to the provocateur. The approach occurred face-to-face, out of sight of the other participants. The same participants were again covertly invited to complete further acts in the third and fourth rounds for an additional £20 each time. They were instructed to develop their own method for completing the insider task to avoid raising suspicion of teammates. All participants approached agreed to the insider task. The multiple teams and sequence of rounds provided insiders numerous opportunities to complete their tasks. For example, they could develop friendships with members of other teams for malicious information gathering or distribute their activity across multiple periods to make it more difficult to spot patterns of activity. Similarly, breaks taken by co-workers afforded opportunities for players to compromise security.

Investigative tasks increased in complexity throughout the game. Similarly, the insider task increased in complexity. In round 2, insiders were instructed to retrieve information from a database they had legitimate access to but which was irrelevant to their team’s intelligence task. In round 3, to provide information from a database only legitimately accessible by another team member. In round 4, to gather information from a database that was only accessible by members of another team. Once the game was complete, players were informed that there had been a security breech, and that their behaviour during the simulation would be investigated. Each participant was then required to individually complete InSort. All insiders completed each of the insider tasks set.

## Materials

The iCOS software comprised five primary modules: a password-protected database creation module, a player interface, a data/keystroke capture module, an investigator interface and a game configuration module. The software provided an ‘electronic’ footprint of activities undertaken by each player, including searches of particular databases, use of email, use of internet and use of printer for each system user. Footprint data and communication data were used to verify participants’ answers to InSort questions. Players were informed that because they were working in a security sensitive environment they were being monitored at all times. This included digital video recording, keystroke data, and monitoring mobile phone usage (text and voice).

InSort comprised 56 questions of which 16 were repeated (example questions see Appendix A):

- Two questions collected information regarding team membership and role, answered via a drop down menu.
- One question asked participants to indicate which databases they had access to as a function of their role and team, again via a drop down menu.
- Three open target questions invited textual responses regarding incident-specific duties, communications activity and movements around the office including access to the printer room and printing activity.
- Eight forced choice yes/no questions concerned password security and adherence to iCOS rules and regulations regarding data security.

The following yes/no questions were repeated twice, randomly throughout the InSort interview:

- Four related to access to each of the four databases.
- Four concerned attempted (but unsuccessful) access to each of the four databases. Four concerned mobile phone usage (1), SMS messaging (1), emailing documents (1) and email behaviour (1).
- Four questions concerned visiting the meeting room, meeting other players, visiting the printer room and printer use.

Participants received instructions on completing InSort, after which they logged in using a unique identifier. Participants could only move forwards through InSort and were unable to skip questions. On completion, participants provided feedback regarding player strategies, behaviours and perceptions of InSort via a hard copy questionnaire comprising 10 questions with Likert scale (ranging from 1 to 5) or yes/no responses.

This research complied with the American Psychological Association Code of Ethics and was approved by the Lancaster University Institutional Review Board. Informed consent was obtained from each participant (materials are available from the first author).

## Results

### Duration (H^1^)

Two-way ANOVAs revealed a significant main effect of group (insider, non-insider), *F* (1, 54) = 187.81 *p* < .001, *η*_*p*_^*2*^ = 0.88. Insiders took twice as long to complete InSort (*M* = 696s, SD = 120.28, 95% CI, 626.62; 765.52) than non-insiders (*M* = 340s, SD = 79.37, 95% CI, 316.15; 363.29). Main effects of team (Narcotics, Fraud, Trafficking) and team role (Administrator, Field Agent, Intelligence Analyst, Tactical Investigator) and all interactions were non-significant, as were the all *F*s < 0.35, all *p*s > .097.

### Word Count and Information Content (H^2^)

**Figure 1.**
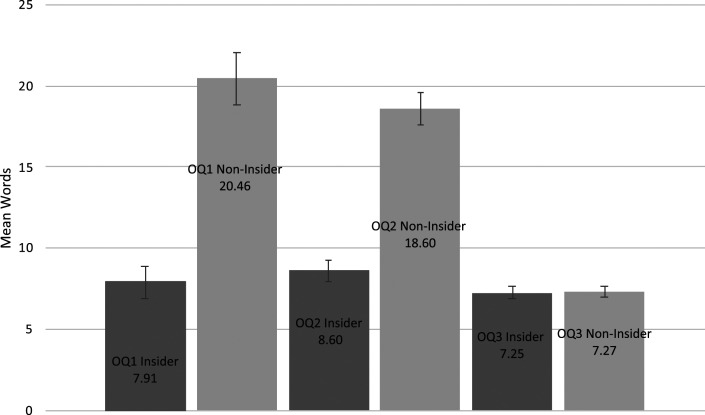
Mean word count for each of open question (OQ1, OQ2 and OQ3) as a function of group (insider; non-insider).

Two-way ANOVAs revealed a significant main effect of group (insider, non-insider) for the total number of words in response to each open target questions, *F* (1, 36) = 12.866, *p* = .001, *η*_*p*_^*2*^ = 0.26, and *F* (1, 36) = 23.95, *p* < .001, *η*_*p*_^*2*^ = 0.40, respectively (see Fig. 1). Non-insiders wrote three times more words (SD = 10.67) than insiders (SD = 2.21) for OQ1 and 2.5 more words (SD = 18.43) for OQ2 than insiders (SD = 8.40). Main effects of team (Narcotics, Fraud, Trafficking) and team role (Administrator, Field Agent, Intelligence Analyst, Tactical Investigator) were non-significant, as were all interactions, all *p*s > .554 (see Table 1). OQ3 was only available to participants who responded ‘yes’ to questions concerning printer usage, emailing documents for printing and visiting the printer room. Accordingly, 25 participants responded to OQ3, of which seven were insiders (50% of insiders; 30% of non-insiders). A one-way ANOVA revealed no significant difference between insiders and non-insiders for total word count in response to OQ3, *p* = .894 (see Fig. 1).

**TABLE 1:** Mean information items for each open question (OQ1, OQ2 and OQ3) as a function of group (insider; non-insider)

	Insider	Non-Insider
	M (95% CI)	
Open question 1	0.86 (.31: 1.41)	4.30 (3.43: 5.18)
Open question 2	1.07 (.50: 1.65)	3.85 (3.43: 4.26)
Open question 3	.86 (.22: 1.50)	1.50 (1.11: 1.89)

Information items in response to open target questions (OQ1, OQ2 and OQ3) were calculated by summing the number of correct, discrete, quantifiable investigation relevant information (IRI) items (see Oxburgh et al., 2012: Phillips et al., 2012 for more on IRI). For example, the following response was coded as six information items, *‘Over the day I was tasked with looking at conversations* ^*1*^ *and other intelligence information in the human trafficking intercepts database* ^*2*^*. I did this to try and track down and formulate an arrest list* ^*3*^ *for the leaders of the Zebra gang* ^*4,*^ *the Garfunkels gang* ^*5*^ *and by working in collaboration with my team members, particularly the tactical investigator* ^*6*^*”*.

Responses to open questions were initially coded by a researcher naïve to the research design and hypotheses following a set of guidelines. 20% (12) of responses from each of the three questions (randomly selected) then underwent independent secondary coding. Inter-rater agreement (IRA) between the coders was high for each of the open questions, *r* = .916 (OQ1), *r* = .882 (OQ2) and *r* = .902 (OQ3).

Two-way ANOVAs revealed significant main effects of group for total information items in OQ1 (individual roles) and OQ2 (individual movements), *F* (1, 36) = 9.485, *p* = .003, *η*_*p*_^*2*^ = 0.22 and, *F* (1, 36) = 34.75, *p* < .001, *η*_*p*_^*2*^ = 0.49, respectively. No other main effects nor interactions emerged, all *p*s > .071. In response to OQ1 and OQ2, insiders provided far less information than non-insiders (see Table 1).

### Closed target question errors (H^3^)

Answers to each of the questions that comprised the four clusters of closed repeated target questions were scored as correct (awarded 1) or incorrect (awarded 2) at Time 1 (first presentation) and in a similar fashion again at Time 2 (second presentation) resulting in an overall target question consistency score for each participant (lower score indicates fewer errors) per cluster (see Table 2). Answers were scored as correct only if participants responded in accordance with behaviours known to match the electronic footprint and surveillance data. The maximum error score (answered incorrectly at Time 1 & 2) was 16. A score of eight indicated respondents were correct on both occasions.

**TABLE 2:** Mean target question cluster error scores a function of group (insider; non-insider) where, max. error score = 16, min. = 8

M (95% CI)	Insider	Non-Insider
Database accessed	9.93 (9.07: 10.79)	9.45 (9.08: 9.88)
Database access attempted	10.43 (9.26: 11.60)	9.52 (9.09: 9.95)
Communication	12.36 (11.15: 13.57)	9.50 (9.05: 9.95)
Movement	8.71 (8.19: 9.24)	8.78 (8.05: 9.03)

Two-way ANOVAs revealed non-significant effects of group, team and team role and non-significant interactions for successful database access target questions, all *p*s > .131. Similarly, target question scores for attempted database access revealed non-significant main effects and interactions, all *p*s > .077. A significant main effect of group (insider, non-insider) emerged for target question scores for communication behaviours, *F* (1, 36) = 29.268, *p* < .001, *η*_*p*_^*2*^ = 0.45. Insider’s scored higher than non-insiders’, indicating discrepancies in responding. All other main effects and interactions were non-significant, all *p*s > .103. Target question scores for the cluster of movement questions revealed non-significant main effects and interactions, all *p*s > .168.

### Answer-Evidence Inconsistency (H^3^)

Answers to closed target questions at Time 1 were scored as consistent (1) or inconsistent (2) with known evidence. Scores were summed, referred to as the *answer-evidence inconsistency scale*, where a lower score indicates higher answer-evidence consistency. Mann-Whitney tests (data violated parametric assumptions) revealed a significant difference between insiders and non-insiders for answer-evidence inconsistency scores, *U* = 43.00, z = −5.046, *p* < .001, *r* = −.084. Overall, insiders’ answer-evidence inconsistency scores were higher (*Mdn* = 22.07) than non-insiders’ (*Mdn* = 15.85).

### Post InSort Feedback (H^4^)

All participants reported understanding the InSort instructions and complying with instructions. Insiders (*M*_Insiders_ =1.93, 95% CI, 1.51: 2.35) reported InSort more demanding (*M*_Non-Insiders_ = 3.52, 95% CI, 3.27: 3.78), *F* (1, 59) = 39.11, *p* < .001, *η*_*p*_^*2*^ = 0.67, and were less confident that their answers were correct, *F* (1, 59) = 45.41, *p* < .001, *η*_*p*_^*2*^ = 0.62 (*M*_Insiders_ = 4.29, 95% CI, 3.52: 5.05 and *M*_Non-insiders_ = 2.04, 95% CI, 1.74, 2.34). Insiders reported finding the questions more difficult, *F* (1, 59) = 7.22, *p* = .009, *η*_*p*_^*2*^ = 0.41 (*M*_Insiders_ = 1.50, 95% CI, 1.20: 1.80 and *M*_Non-insiders_ = 2.11, 95% CI, 1.87, 2.17).

Insiders reported being dishonest when answering questions, *X*^2^ (1, 60) = 19.543, *p* < .001 and withholding information more often, *X*^2^ (1, 60) = 24.65, *p* < .001. There was no difference between insiders and non-insiders when asked whether InSort questions had been repeated, *p* = .634. Overall, 27 responded (45%) ‘no’, 16 (27%) were ‘unsure’ and 17 (28%) said ‘yes’. Again, no difference emerged between insiders and non-insiders as which types of questions (yes/no or text responses) had been more demanding, *p* = .370. Overall, 25 (42%) reported yes/no questions to be most demanding, 26 (43%) textual responses, and 9 (15%) reported all questions were equally demanding.

## Discussion

Insider attacks are increasing in number and magnitude, with potential to undermine national and international security, cause financial loss and reputational damage (e.g., Legg, 2017; Wei et al., 2021). We developed InSort, a text-based synchronous triage with potential for highlighting persons of interest after an insider incident. Insiders took twice as long to complete InSort, were less confident their answers were correct, found InSort more cognitively challenging, provided less information, and typed fewer words. Our results confirm findings of previous research in face-to-face and remote person-to-person contexts that questioning strategies which maximize cognitive burden can amplify signals of deception (e.g., Bogaard et al., 2016; DePaulo et al., 2003; Pentland et al., 2017), highlighting the potential of remote automated CMC.

Open questions increased the information harvested, eliciting an individual’s version of the truth, which can be explored for veracity (e.g., Kontogianni et al., 2020; Snook et al., 2010). Tactical questioning, concerning known or verifiable information are spread throughout InSort rather than clustered at the beginning or end, which improves the veracity performance by interviewers and observers (Dando et al., 2015; Levine, 2018). We incorporated both where response time was not constrained, but where response time was monitored. Yet, although respondents could take their time and did not have to consider social context and how their answers/behaviours were received, again tactical questioning leveraged diagnostic indicators across a cohort.

The remote CMC nature of InSort may have diverted impression management towards behaviours perceived by insiders as more important, hence engendering differences in the time taken to complete InSort and in the informational content in open question responses. The absence of a human questioner, and without understanding the importance of *all* response behaviours, some behaviours were attended to at the expense of others. Providing a coherent and consistent narrative without contradictions, with little time to prepare and where questions are not chronologically ordered, may explain the increased duration. Insider responses to open target questions were shorter, suggesting they were seeking to appear credible and cooperative, simultaneously being cautious in responding (see Schuetzler et al., 2019; Sporer, 2016; Zuckerman et al., 1981). Wordy replies with low information can be indicative of deception, but not always. However, here short information poor replies were indictive of insiders, possibly being deceptive by withholding information, which is reported in face-to-face contexts (De Rosa et al., 2019; Levine, 2018)

Our findings are consistent with findings regarding the efficacy of automated screening systems for detecting deception at border crossings and in job interviews, further indicating that textual response content and response behaviours are important (see Chattopadhyay et al., 2018; Nunamaker et al., 2011; Schuetzler, et al., 2019). Our results are also consistent with cognitive load explanations of deceptive communication (Fenn et al., 2015; Ho et al., 2016). Creation and then typing of answers to questions is complex and time consuming, but the additional demands associated with being deceptive is more time consuming still. Deceptive textual communications are shorter due to the challenges of drawing multiple responses from memory as plausible answers to questions (e.g., Burgoon et al., 2003; Pollina et al., 2017; Schuetzler et al., 2019).

Manipulative questioning includes repeat questions, which we believed could leverage notable inconsistencies between insiders and non-insiders because insiders would struggle to provide credible and consistent responses to repeat questions (H^3^). Our question cluster scores alone did not generally support this hypothesis. However, one important finding was that insiders did not successfully monitor their communication behaviour and so were unable to maintain consistency. Future triage approaches might consider capturing detailed human-human remote interaction behaviours.

Although the consistency across time literature in face-to-face contexts is mixed, our findings suggest deceivers can be as consistent, sometimes more so than truthtellers (e.g., Blair et al., 2018; Clemens & Grolig, 2019; Masip et al., 2018). Conversely, answer-evidence inconsistency scores differed significantly. While insiders were consistent in textual responses, responses to target questions were inconsistent with evidence, which mirrors results in face-to-face contexts (Hartwig et al., 2006; Sukumar et al., 2018). However, here participants were aware their behaviour was monitored throughout and that movement information was collected. In face-to-face contexts participants are often unaware of information known by interviewers, which is fundamental to the success of tactical and strategic interviewing techniques (e.g., see Oleszkiewicz & Watson, 2021). Here, despite knowing behaviour information was collected, answer-evidence inconsistency again emerges as a useful metric with potential for improving veracity decisions.

Information Manipulation Theory 2 (McCornack et al., 2014: IMT2) may be relevant whereby cognitive load is related to difficulty of reasoning through the problem space created by a gap between the initial state, in our study the questions asked by InSort, and the end state (avoidance of detection). IMT2 suggests lies are produced only when the production of the truth is problematic, and that high cognitive load is not intrinsic to deceptive discourse but depends on the potential number of solutions needed to present the version judged most appropriate. Our game was designed to mimic demands experienced by insiders in a secure environment. Hence, there were numerous narratives insiders could choose. IMT2 also proposes quantity violations such as omitting problematic discourse as a frequent form of deceptive discourse. This might explain why insiders produced fewer words.

### Limitations and Future Directions

Our simulation embodied some features of organizations, but there are differences between it and the real world. As Taylor et al. (2013) point out the absence of a ‘world’ outside the simulation as a limitation. Employees often communicate with individuals outside their own organization, increasing the heterogeneity of communication and collaborative behaviours. Insiders were chosen at random without controlling/measuring personality, motivation or personal circumstances, which may not tally with how insiders emerge. More complex simulations could manage these variables. We compared known insiders to co-workers as a first step towards understanding if InSort might leverage differences in textual responses with reference to theories of cognitive load, information manipulation and deception. More research is required to understand how to delineate signal from noise where status is unknown. Finally, the structure of InSort is guided by the applied deception literature and so likely to remain fairly consistent. However, the informational content of questions is dynamic. Ours was bespoke to the iCOS simulation. Constructing an event-specific InSort triage depends upon the nature of tasks workers are required and allowed to do day-to-day, the information known to employers, and the insider event itself, which would guide the informational content.

## Conclusions

Findings demonstrate the potential of real time remote investigative triage approaches such as InSort. InSort could regularly be implemented on an ad hoc basis as part of in-house security practices following operations or investigations of the nature described here. This may be useful for collating databases of response behaviours such as answer lengths and response times. Such a database may offer additional information alongside the event-specific ‘footprint’ allowing comparisons across incidents. InSort can be constructed and administered by non-specialists and quickly altered as required across incidents. As such, InSort has potential to expedite investigative processes.

## Appendix A: Example InSort Questions

Example open question:

1. *‘Please explain what your team role entailed’* Answer via free textual response

Example closed non-target questions:

1. *‘What team were you assigned too?’* Answer via a forced choice (one choice allowed) drop down menu
2. *‘What was your role in the team?’* Answer via a forced choice (one choice allowed) drop down menu

Example closed target questions (multiple responses option):

1. *‘Which databases did your team role allow you to access?’* Answer via a drop down menu allowing multiple choices
2. *‘Which data bases did you access during the investigation?’* Answer via a drop down menu allowing multiple choices

Example closed target questions (forced choice response):

1. *‘Did you attempt to access the ‘Shared Network’ database?’* Yes/no
2. *Did you share your ‘Shared Network’ database password with anyone?’* Yes/no
